# Use of the Advanced Lung Cancer Inflammation Index as a Prognostic Indicator for Patients With Cholangiocarcinoma

**DOI:** 10.3389/fsurg.2022.801767

**Published:** 2022-01-27

**Authors:** Huasheng Wu, Fadian Ding, Meitai Lin, Zheng Shi, Zhengzhou Mei, Shaoqin Chen, Chao Jiang, Huabin Qiu, Zhenhua Zheng, Youting Chen, Peng Zhao

**Affiliations:** ^1^Department of Hepatobiliary Surgery, San Ming First Hospital, Sanming, China; ^2^Department of Hepatobiliary Surgery, The First Affiliated Hospital of Fujian Medical University, Fuzhou, China; ^3^Department of Intensive Care Unit, Xiamen Cardiovascular Hospital, Xiamen University, Xiamen, China; ^4^Department of Gastroenterology, San Ming First Hospital, Sanming, China

**Keywords:** cholangiocarcinoma, prognosis, advanced lung cancer inflammation index nomogram, NLR, MLR, SII

## Abstract

**Background:**

This study aimed to assess the clinical utility of the advanced lung cancer inflammation index (ALI) as a prognostic indicator for patients with cholangiocarcinoma (CCA) and construct a prognostic nomogram based on ALI.

**Methods:**

A total of 97 CCA patients who received radical resection were included. The optimal cut-off point for ALI was identified by X-tile analysis. COX regression analysis were used to identify risk factors of overall survival (OS) and disease-free survival (DFS). A predictive nomogram for DFS was constructed.

**Results:**

The optimal cut-off value for preoperative ALI was 31.8. 35 (36.1%) patients were categorized into the low-ALI group and 62 (63.9%) patients into the high-ALI group. Low ALI was independently associated with hypoproteinemia and lower body mass index (BMI) (all *P* < 0.05). COX regression analysis revealed that preoperative ALI level (HR = 0.974, *P* = 0.037) and pathological TNM stage (HR = 7.331, *P* < 0.001) were independently correlated with OS for patients with CCA, and preoperative ALI level (HR = 0.978, *P* = 0.042) and pathological T stage (HR = 1.473, *P* = 0.035) remained to be independent prognostic factors for DFS in CCA patients. Using time-dependent ROC analysis, we found that ALI was better at predicting prognosis than other parameters, such as neutrophil-to-lymphocyte ratio (NLR), monocyte-to-lymphocyte ratio (MLR), platelet-to-lymphocyte ratio (PLR), systemic immune-inflammation index (SII), and prognostic nutritional index (PNI) in terms of OS and DFS. A nomogram predicting DFS was built (C-index: 0.73 95%CI: 0.67–0.79).

**Conclusions:**

ALI may be useful for prognosis assessment for patients with CCA.

## Introduction

Cholangiocarcinoma (CCA) is a devastating biliary malignancy in the biliary tree with an increasing incidence worldwide (1, 2). The mainstay of treatment of CCA includes surgery, locoregional and systemic therapies (3). However, the prognosis of CCA patients remains dismal, mainly owing to the advanced tumor stage at diagnosis and high tumor relapse rates (4). The widely-used tumor-node-metastasis (TNM) staging system depends on the locoregional tumor expansion of the primary tumor and thus ignores the biological differences between tumor and host (5). Therefore, identifying valid prognostic factors of CCA is highly needed to facilitate personalized therapy guidance and improve the prediction of patient prognosis.

Systemic inflammation is vital in tumor development and progress in several cancers (6), including CCA (7). Previous studies have demonstrated that serum systemic inflammatory markers, including neutrophil-to-lymphocyte ratio (NLR), platelet-to-lymphocyte ratio (PLR), systemic immune-inflammation index (SII), and can help predict the survival of CCA patients (8–10). In addition, nutritional status is also helpful for predicting the prognosis of CCA (11). Advanced lung cancer inflammation index (ALI) is a novel inflammation and nutritional index by combining body mass index (BMI), preoperative serum albumin, and NLR (12). In recent years, ALI has been proposed as a prognostic biomarker of several cancers, such as lung (13), colorectal (14), esophageal (15), and gastric (16) cancers. To our knowledge, the clinical value of ALI in patients with CCA has not yet been studied.

We hypothesized that ALI could be a candidate prognostic indicator for CCA patients. In this context, we aimed to explore the clinical implication of ALI as a prognostic predictor of patients with CCA. Based on ALI, we further established a nomogram for predicting disease-free survival (DFS).

## Materials and Methods

### Patients

This study was a retrospective analysis of 97 consecutive patients with CCA who underwent radical surgery between 2016 and 2019 at the Department of Hepatobiliary Surgery, The First Affiliated Hospital of Fujian Medical University. The inclusion criteria were as follows: (1) pathologically confirmed CCA, (2) patients who underwent primary radical resection, and (3) aged > 18 years. Exclusion criteria included: (1) evidence of recurrent or metastatic disease, (2) preoperative anti-tumor treatment, (3) palliative or non-radical resection, (4) coexistent hematological disorders, and (5) infection before treatment. The baseline clinicopathological characteristics of patients were reviewed retrospectively from the medical records, and the patient's informed consent was waived. The study protocol was approved by the Institutional Review Board of The First Affiliated Hospital of Fujian Medical University (NO. 2018-053).

### Treatment and Follow-Up

The routine preoperative assessment was performed to assess the tumor resectability, including general condition, physical examination, important organ function (heart, lung, liver, and kidney, etc.), chest X-ray or computed tomography (CT) scans, abdominal ultrasound or CT scans. Depending on the tumor's location, hepatic resection was performed for patients with intrahepatic CCA, hepatic resection with segmental bile duct resection for hilar CCA, and pancreaticoduodenectomy for patients with distal CCA.

Pathological staging of CCA was performed according to the 8th edition of the American Joint Committee on Cancer (AJCC) staging systems (17). Postoperative follow-up was regularly performed, including physical examination, serum carbohydrate antigen 19-9 (CA19-9), α-fetoprotein (AFP), carcinoembryonic antigen (CEA), abdominal ultrasound, or CT scan, and chest X-ray or CT scan every 3 to 6 months. Overall survival (OS) was calculated as surgery to death or the last follow-up. DFS was determined as the time from surgery to tumor relapse. Patient follow-up lasted until death or the cut-off date of August 1, 2021.

### Laboratory Assays

All laboratory data were obtained within 1 week before surgery. The formulas were as follows: NLR = neutrophil count (10^9^/L) / lymphocyte count (10^9^/L), PLR = platelet count (10^9^/L) / lymphocyte count (10^9^/L), MLR = monocyte count (10^9^/L) / lymphocyte count (10^9^/L). ALI = BMI (kg/m^2^) × albumin (g/L) /NLR, SII = platelet count (10^9^/L) × neutrophil count (10^9^/L) / lymphocyte count (10^9^/L), and prognostic nutritional index (PNI) = serum albumin (g/L) + 5 × lymphocyte count (10^9^/L).

### Statistical Analysis

All statistical analyses were carried out using IBM SPSS 24.0 (IBM SPSS Statistics, Chicago, IL, USA) and R 3.6.1 (http://www.R-project.org/). The X-tile program was used to determine the optimal cut-off value for ALI, NLR, MLR, PLR, SII, and PNI based on DFS. When appropriate, categorical variables were compared with the *Chi-square* test (two-tailored). The Student's *t*-test was used to compare continuous variables between groups. The Kaplan-Meier method and the log-rank test were used to estimate survival between groups. Cox proportional hazards models were used to evaluate the prognostic value of the ALI. A nomogram predicting DFS was constructed based on the independent predictors in the Cox regression model. The performance of the nomogram was evaluated by Harrell's C-index and internally validated using the bootstrap method. The prognostic efficacy of different models was assessed by time-dependent receiver operating characteristic (time-dependent ROC) analysis. Finally, decision curve analysis (DCA) was performed to determine the clinical usefulness of the nomogram by quantifying the net benefits. *P* < 0.05 was considered statistically significant.

## Results

### Patient Characteristics

A total of 97 patients with CCA were eligible for this analysis. Of them, 58 (59.8%) patients were male, and the mean age was 60.4 ± 10.4 years. The baseline clinicopathological characteristics of patients are presented in Table 1. As seen in Supplementary Figure 1, X-tile plots identified 31.8 as cut-off values for preoperative ALI. Accordingly, 2.9, 0.3, 94.2, 682.4, and 46.8 were determined as optimal cut-off values for preoperative NLR, MLR, PLR, SII, and PNI in patients with CCA, respectively. As shown in Supplementary Table 1, the baseline characteristics did not differ significantly between tumor locations of CCA.

**Table 1:** Baseline characteristics in patients with cholangiocarcinoma stratified by ALI.

**Characteristics**	**Total (*n =* 97)**	**ALI <31.8 (*n =* 35)**	**ALI ≥31.8 (*n =* 62)**	***P-*value**
Sex (%)				0.089
Male	58 (59.8)	25 (71.4)	33 (53.2)	
Female	39 (40.2)	10 (28.6)	29 (46.8)	
Age (years)	60.4 ± 10.4	58.8 ± 11.3	61.2± 9.8	0.268
ASA score (%)				0.466
1	62 (63.9)	25 (71.4)	37 (59.7)	
2	30 (30.9)	9 (25.7)	21 (33.9)	
3	5 (5.2)	1 (2.9)	4 (6.5)	
Preoperative CEA level (%)				0.258
<5.0 ng/mL	31 (32.0)	14 (40.0)	17 (27.4)	
≥ 5.0 ng/mL	66 (68.0)	21 (60.0)	45 (72.6)	
Preoperative CA19-9 level (%)				0.125
<37.0 U/mL	36 (37.1)	9 (25.7)	27 (43.5)	
≥ 37.0 U/mL	61 (62.9)	26 (74.3)	35 (56.5)	
Preoperative AFP level (%)				0.504
<25.0 μg/L	33 (34.0)	10 (28.6)	23 (37.1)	
≥ 25.0 μg/L	64 (66.0)	25 (71.4)	39 (62.9)	
Anemia (%)	28 (28.9)	13 (37.1)	15 (24.2)	0.243
Hypoproteinemia (%)	18 (18.6)	11 (31.4)	7 (11.3)	**0.027**
CRP (%)				0.161
<10.0 mg/L	87 (89.7)	29 (82.9)	58 (93.5)	
≥ 10.0 mg/L	12(10.3)	6 (17.1)	4 (6.5)	
PCT (%)				**0.032**
<0.06 ng/mL	83 (85.6)	26 (74.3)	57 (91.9)	
≥0.06 ng/mL	14 (14.4)	9 (25.7)	5 (8.1)	
BMI (%)				**0.022**
<18 kg/m^2^	2 (2.1)	2 (5.7)	0	
18–24 kg/m^2^	62 (63.9)	26 (74.3)	(58.1)	
≥24 kg/m^2^	33 (34.0)	7 (20.0)	26 (41.9)	
Tumor location				0.848
Peripheral	34 (35.1)	12 (34.3)	22 (35.3)	
Hilar	49 (50.5)	17 (48.6)	32 (51.6)	
Distal	14 (14.4)	6 (17.1)	8 (12.9)	
Tumor differentiation (%)				0.328
Well to moderately differentiated	73 (75.3)	24 (68.6)	49 (79.0)	
Poorly differentiated and others	24 (24.7)	11 (31.4)	13 (21.0)	
Postoperative complications (%)	38 (39.2)	12 (34.3)	26 (42.6)	0.517
Pathological T stage (%)				0.327
1	3 (3.1)	0 (0.0)	3 (4.8)	
2	26 (26.8)	7 (20.0)	19 (30.6)	
3	55 (56.7)	23 (65.7)	32 (51.6)	
4	13(13.4)	5 (14.3)	8 (12.9)	
Pathological N stage (%)				1.000
Negative	56 (57.7)	20 (57.1)	36 (58.1)	
Positive	41 (42.3)	15 (42.9)	26 (41.9)	
Pathological M stage (%)				0.572
0	81 (83.5)	28 (80.0)	53 (85.5)	
1	16 (16.5)	7 (20.0)	9 (14.5)	
Nerval invasion (%)	50 (51.5)	16 (45.7)	34 (54.8)	0.406

*ALI, advanced lung cancer inflammation index; ASA, American Society of Anesthesiologists; CEA, carcinoembryonic antigen; CA19-9, carbohydrate antigen 19-9; AFP, α-fetoprotein; CRP, C-reactive protein; PCT, procalcitonin; BMI, body mass index. Bond fonts indicate of statistical significance*.

### Association of ALI With Clinicopathological Characteristics

Based on the optimal cut-off value, these patients were dichotomized into the low-ALI group (*n* = 35, 36.1%) and the high-ALI group (*n* = 62, 63.9%). No significant differences were found between the groups regarding baseline characteristics, such as sex, age, American Society of Anaesthesiologists (ASA) score, preoperative CEA level, preoperative CA19-9 level, preoperative AFP level, and anemia (all *P* > 0.05, Table 1). As expected, a lower preoperative ALI level was associated with hypoproteinemia and a lower BMI (*P* = 0.027 and *P* = 0.022, respectively). A lower preoperative ALI level was also associated with a lower procalcitonin (PCT) level. Regarding postoperative complications, no significant differences were seen between the two groups in terms of tumor location, tumor differentiation, pathological T stage, pathological N stage, pathological M stage, and nerval invasion (all *P* > 0.05).

### Correlations Between ALI and Survival

**Figure 1:**
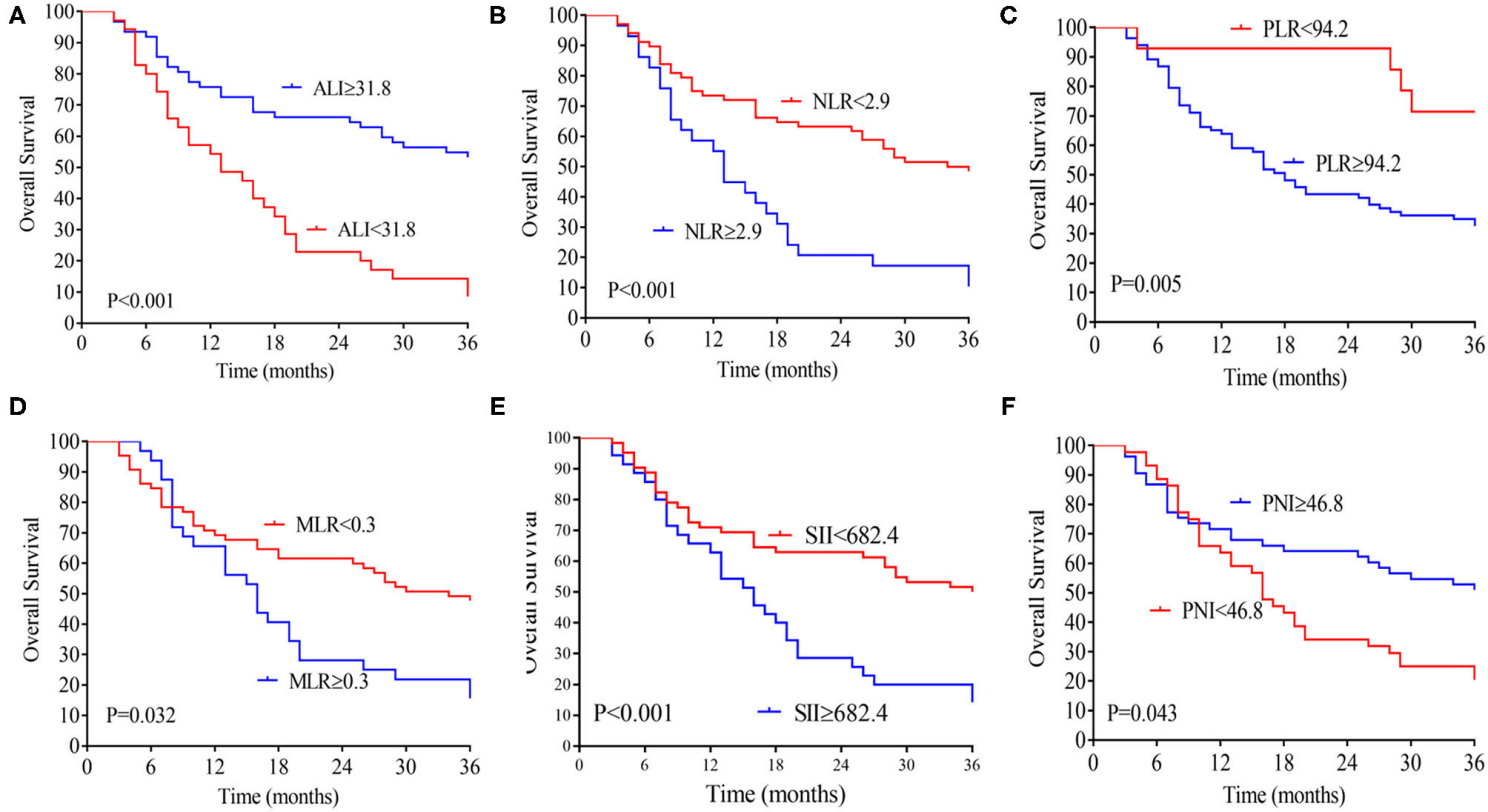
Kaplan-Meier analysis of the ALI **(A)**, NLR **(B)**, MLR **(C)**, PLR **(D)**, SII **(E)**, and PNI **(F)** counts in terms of OS. ALI, advanced lung cancer inflammation index; NLR, neutrophil-to-lymphocyte ratio; MLR, monocyte-to-lymphocyte ratio; PLR, platelet-to-lymphocyte ratio; SII, systemic immune-inflammation index; PNI, prognostic nutritional index; OS, overall survival.

**Figure 2:**
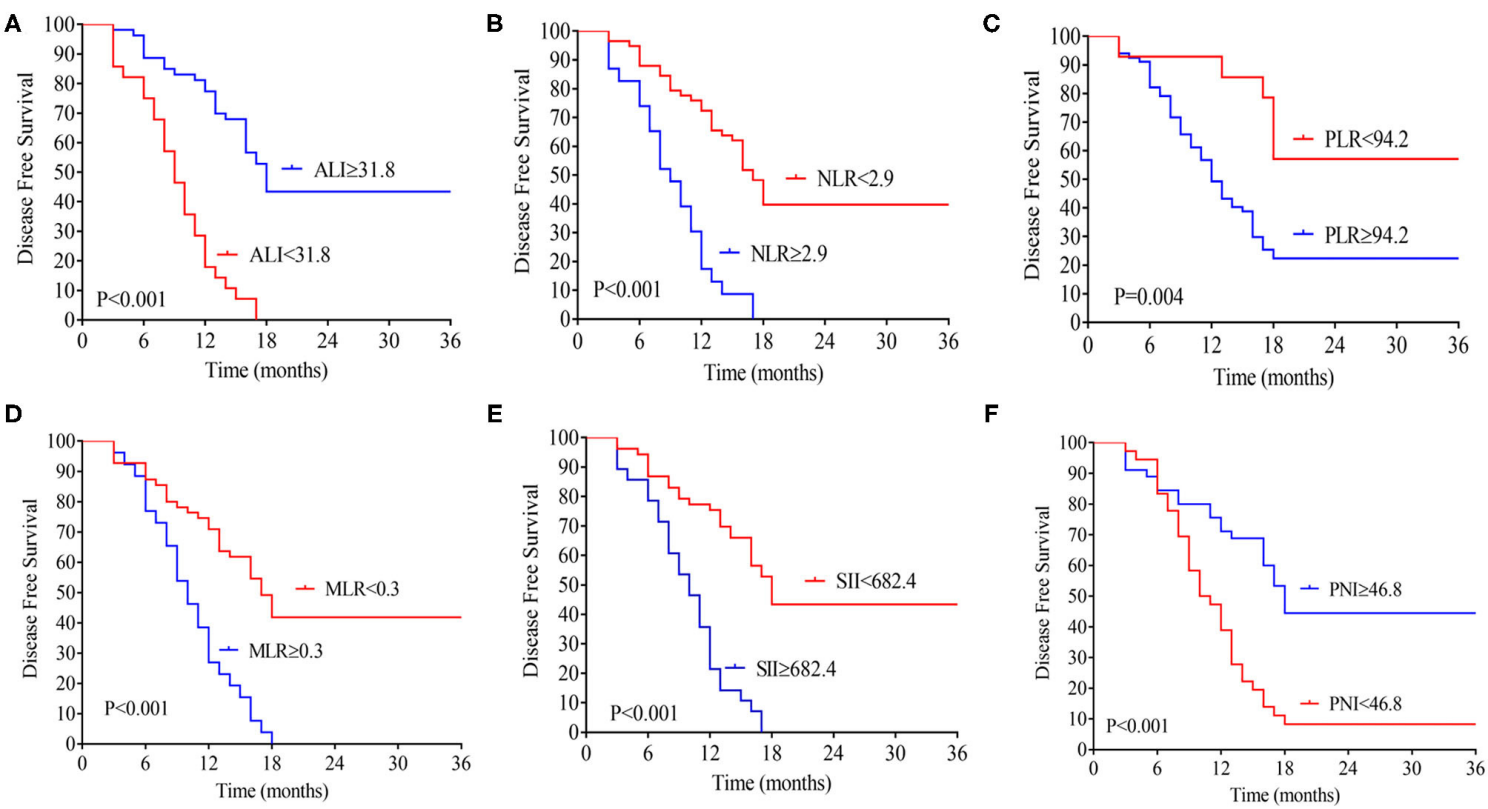
Kaplan-Meier analysis of the ALI **(A)**, NLR **(B)**, MLR **(C)**, PLR **(D)**, SII **(E)**, and PNI **(F)** counts in terms of DFS. ALI, advanced lung cancer inflammation index; NLR, neutrophil-to-lymphocyte ratio; MLR, monocyte-to-lymphocyte ratio; PLR, platelet-to-lymphocyte ratio; SII, systemic immune-inflammation index; PNI, prognostic nutritional index; DFS, disease-free survival.

The median follow-up time was 20 months (range 3–70 months), 74 patients (76.2 %) developed distant metastasis, and 64 patients (65.9%) died after radical resection. Low preoperative level ALI was significantly associated with poorer 3-year OS (**Figure 2A**) and DFS (both *P* < 0.01, **Figure 2B**). The 3-year OS rates in low and high ALI patients were 11.4% and 54.8% (*P* < 0.01), respectively. The 3-year DFS rates in low and high ALI patients were 0 and 43.4% (*P* < 0.01), respectively. We also evaluated the prognostic value of preoperative NLR, PLR, and MLR, SII, and PNI in OS and DFS. As shown in Figures 1, 2, higher preoperative NLR, PLR, MLR, and SII scores were correlated with worse OS and DFS in patients with CCA (all *P* < 0.01). Besides, higher PNI was associated with worse OS and DFS in CCA patients (all *P* < 0.01).

### Cox Regression Analysis of Risk Factors of OS

Univariate analysis revealed that preoperative hypoproteinemia [hazard ratio (HR) = 2.029, 95%CI: 1.143–3.602, *P* = 0.016], preoperative NLR level (HR = 1.224, 95%CI: 1.086–1.380, *P* = 0.001), preoperative SII level (HR = 1.001, 95%CI: 1.000–1.001, *P* = 0.001), preoperative MLR level (HR = 13.414, 95%CI: 2.822–63.759, *P* = 0.001), preoperative ALI level (HR = 0.974, 95%CI: 0.960–0.988, *P* < 0.001), preoperative PNI level (HR = 0.931, 95%CI: 0.888–0.977, *P* = 0.004), tumor differentiation (HR = 1.924, 95%CI: 1.132–3.270, *P* = 0.016), and pathological TNM stage (HR = 1.700, 95%CI: 1.227–2.355, *P* = 0.001) were associated with OS in CCA patients. Multivariate COX regression analysis revealed that preoperative ALI level (HR = 0.974, 95%CI:0.951–0.998, *P* = 0.037) and pathological TNM stage (HR = 7.331, 95%CI: 3.576–15.030, *P* < 0.001) were independently correlated with OS for patients with CCA (Table 2).

**Table 2:** Cox regression analysis of predictive factors for overall survival in patients with cholangiocarcinoma (*n* = 97).

**Variables**	**Univariate analysis**			**Multivariate analysis**		
	**HR**	**95% CI**	***P*-value**	**HR**	**95% CI**	***P*-value**
Sex, male/female	1.310	0.797–2.154	0.286			
Age	0.997	0.974–1.020	0.794			
ASA score	0.801	0.520–1.233	0.313			
BMI	0.667	0.421–1.058	0.086			
Tumor location			0.343			
Peripheral	Reference	Reference	Reference			
Hilar	0.955	0.553–1.650	0.869			
Distal	1.579	0.764–3.266	0.218			
Preoperative AFP level	0.927	0.550–1.564	0.777			
Preoperative CEA level	1.074	0.628–1.836	0.795			
Preoperative CA19-9 level	1.094	0.653–1.834	0.732			
Preoperative anemia	1.002	0.989–1.014	0.788			
Preoperative hypoproteinemia	2.029	1.143–3.602	**0.016**	1.994	0.800–4.966	0.138
Preoperative NLR level (<2.9 vs. ≥2.9)	1.224	1.086–1.380	**0.001**	1.427	0.956–2.130	0.081
Preoperative SII level (<682.4 vs. ≥682.4)	1.001	1.000–1.001	**0.001**	0.999	0.998–1.000	0.149
Preoperative MLR level (<0.3 vs. ≥ 0.3)	13.414	2.822–63.759	**0.001**	0.155	0.007–3.246	0.230
Preoperative PLR level (<94.2 vs. ≥ 94.2)	1.003	1.000–1.006	0.063			
Preoperative ALI level (<31.8 vs. ≥ 31.8)	0.974	0.960–0.988	**<0.001**	0.974	0.951–0.998	**0.037**
Preoperative PNI level (<46.8 vs. ≥ 46.8)	0.931	0.888–0.977	**0.004**	1.046	0.945–1.158	0.382
Preoperative PCT level	1.549	0.809–2.967	0.187			
Preoperative CRP level	1.119	0.510–2.457	0.779			
Tumor differentiation	1.924	1.132–3.270	**0.016**	1.131	0.613–2.086	0.694
Pathological TNM stage	1.700	1.227–2.355	**0.001**	7.331	3.576–15.030	**<0.001**
Nerval invasion	1.054	0.645–1.721	0.835			
Postoperative complications	0.877	0.531–1.450	0.610			
Postoperative hospital stays	0.983	0.957–1.010	0.224			

*HR, hazard ratio; CI, confidential interval; ASA, American Society of Anesthesiologists; CEA, carcinoembryonic antigen; CA19-9, carbohydrate Antigen 19-9; NLR, neutrophil-to-lymphocyte ratio; SII, systemic immune-inflammation index; MLR, monocyte-to-lymphocyte ratio; PLR, platelet-to-lymphocyte ratio; ALI, advanced lung cancer inflammation index; PNI, prognostic nutritional index; PCT, procalcitonin; CRP, C-reactive protein; AFP, α-fetoprotein. Bold fonts indicate of statistical significance*.

### Cox Regression Analysis of Risk Factors of DFS

On univariate analysis, preoperative hypoproteinemia (HR = 2.169, 95%CI: 1.262–3.728, *P* = 0.005), preoperative NLR level (HR = 1.307, 95%CI: 1.156–1.477, *P* < 0.001), preoperative SII level (HR = 1.001, 95%CI: 1.001–1.001, *P* < 0.001), preoperative MLR level (HR = 34.450, 95%CI: 7.096–167.240, *P* < 0.001), preoperative PLR level (HR = 1.005, 95%CI: 1.002–1.007, *P* = 0.002), preoperative ALI level (HR = 0.970, 95%CI: 0.957–0.983, *P* < 0.001), preoperative PNI level (HR = 0.911, 95%CI: 0.870–0.954, *P* < 0.001), pathological T stage (HR = 1.700, 95%CI: 1.227–2.355, *P* = 0.001), and pathological N stage (HR = 1.732, 95%CI: 1.095–2.740, *P* = 0.019) were associated with DFS in CCA patients. After adjustment for confounding factors, multivariate COX regression analysis demonstrated that preoperative ALI level (HR = 0.978, 95%CI: 0.957–0.999, *P* = 0.042) and pathological T stage (HR = 1.473, 95%CI: 1.027–2.111, *P* = 0.035) remained to be independent prognostic factors for DFS in CCA patients (Table 3).

**Table 3:** Cox regression analysis of predictive factors for disease-free survival in patients with cholangiocarcinoma (*n* = 97).

**Variables**	**Univariate analysis**			**Multivariate analysis**		
	**HR**	**95% CI**	***P*-value**	**HR**	**95% CI**	***P*-value**
Sex, male/female	1.203	0.758–1.910	0.433			
Age	0.997	0.976–1.019	0.775			
ASA score	0.824	0.556–1.221	0.334			
BMI	0.658	0.426–1.016	0.059			
Tumor location			0.282			
Peripheral	Reference	Reference	Reference			
Hilar	1.032	0.620–1.716	0.904			
Distal	1.688	0.844–3.375	0.139			
Preoperative AFP level	0.961	0.590–1.564	0.872			
Preoperative CEA level	1.054	0.640–1.736	0.837			
Preoperative CA19-9 level	1.038	0.644–1.674	0.878			
Preoperative anemia	0.996	0.985–1.007	0.501			
Preoperative hypoproteinemia	2.169	1.262–3.728	**0.005**	1.211	0.499–2.935	0.672
Preoperative NLR level (<2.9 vs. ≥ 2.9)	1.307	1.156–1.477	**<0.001**	0.881	0.604–1.286	0.512
Preoperative SII level (<682.4 vs. ≥ 682.4)	1.001	1.001–1.001	**<0.001**	1.000	0.999–1.002	0.518
Preoperative MLR level (<0.3 vs. ≥ 0.3)	34.450	7.096–167.240	**<0.001**	4.470	0.287–78.308	0.277
Preoperative PLR level (<94.2 vs. ≥ 94.2)	1.005	1.002–1.007	**0.002**	1.000	0.994–1.006	0.937
Preoperative ALI level (<31.8 vs. ≥ 31.8)	0.970	0.957–0.983	**<0.001**	0.978	0.957–0.999	**0.042**
Preoperative PNI level (<46.8 vs. ≥ 46.8)	0.911	0.870–0.954	**<0.001**	0.996	0.905–1.096	0.937
Preoperative PCT level	1.670	0.914–3.048	0.095			
Preoperative CRP level	1.111	0.533–2.315	0.779			
Tumor differentiation	1.594	0.952–2.669	0.076			
Pathological T stage	1.700	1.227–2.355	**0.001**	1.473	1.027–2.111	**0.035**
Pathological N stage	1.732	1.095–2.740	**0.019**	1.431	0.879–2.329	0.150
Nerval invasion	1.082	0.686–1.708	0.735			
Postoperative complications	0.835	0.522–1.336	0.835			
Postoperative hospital stays	0.991	0.967–1.016	0.476			

*HR, hazard ratio; CI, confidential interval; ASA, American Society of Anesthesiologists; CEA, carcinoembryonic antigen; CA19-9, carbohydrate Antigen 19-9; NLR, neutrophil-to-lymphocyte ratio; SII, systemic immune-inflammation index; MLR, monocyte-to-lymphocyte ratio; PLR, platelet-to-lymphocyte ratio; ALI, advanced lung cancer inflammation index; PNI, prognostic nutritional index; PCT, procalcitonin; CRP, C-reactive protein; AFP, α-fetoprotein. Bold fonts indicate of statistical significance*.

### Subgroup Kaplan-Meier Analysis of ALI According to the Location of CCA

As shown in Supplementary Figures 2A,D, higher ALI was associated with higher DFS and OS rates for intrahepatic CCA patients (*P* = 0.004, *P* = 0.003, respectively). Similarly, higher ALI was associated with higher DFS and OS rates for hilar CCA patients (both *P* < 0.001, respectively), as depicted in Supplementary Figures 2B,E. However, ALI was not significantly associated with DFS or OS rates in distal CCA patients (*P* = 0.161, *P* = 0.436, respectively), as demonstrated in Supplementary Figures 2C,F.

### Comparison of Prediction Efficiency of ALI and Other Parameters

**Figure 3:**
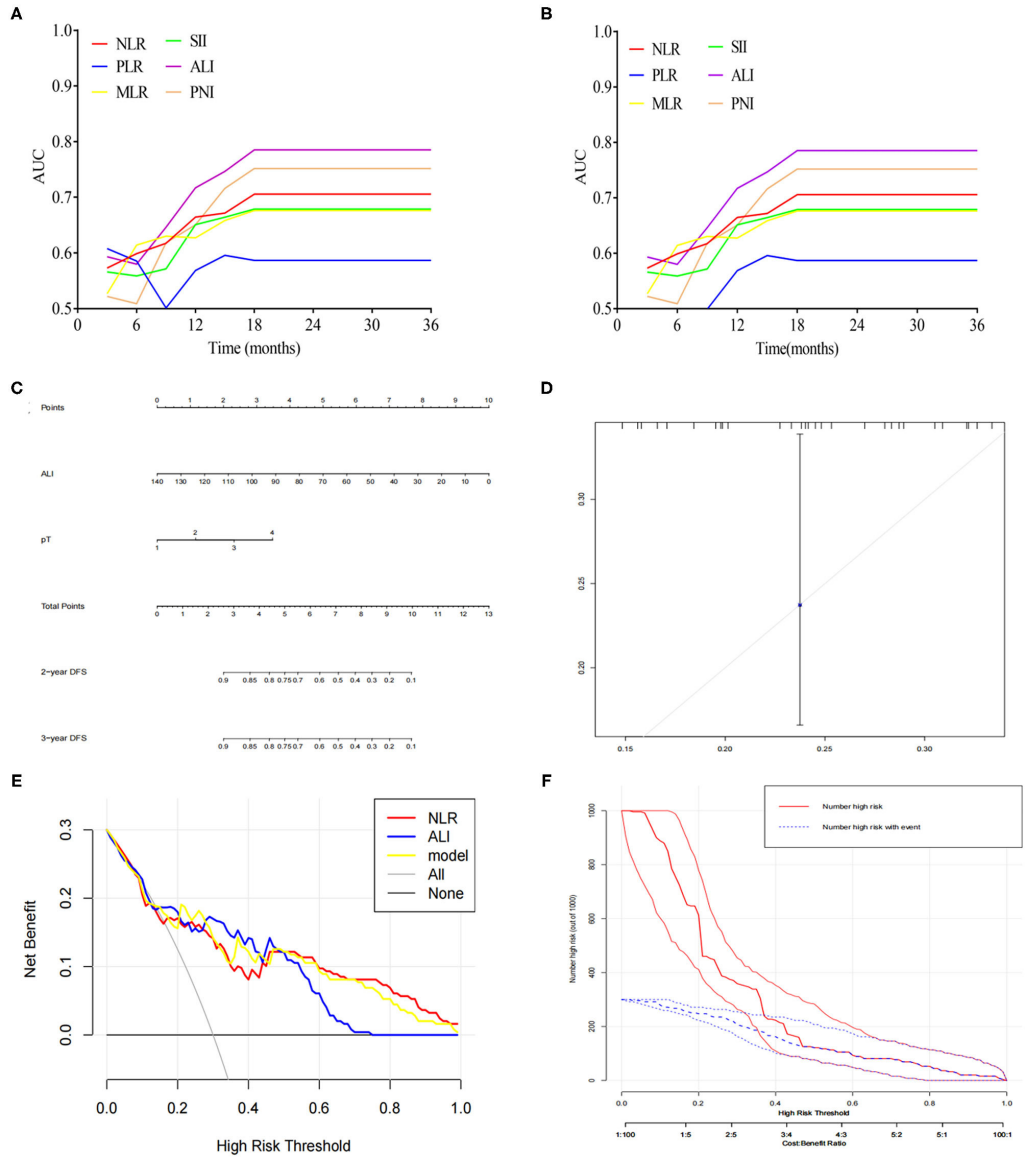
**(A)** Time-dependent ROC analysis of ALI, NLR, MLR, PLR, SII, and PNI for predicting OS. **(B)** Time-dependent ROC analysis of ALI, NLR, MLR, PLR, SII, and PNI for predicting DFS. **(C)** A nomogram was developed for predicting DFS based on ALI. **(D)** Calibration curves for DFS for the nomogram with internal validation. **(E)** Decision curve analysis for ALI. **(F)** Clinical impact curve for the risk model.

By using time-dependent ROC analysis, the prognostic prediction efficiency of ALI was assessed against other parameters. As shown in Figure 3A, the performance of ALI in predicting OS was superior to other parameters, including NLR, PLR, MLR, SII, and PNI during the observation period. Similarly, time-dependent ROC curves revealed that the prediction efficiency of ALI in DFS was superior to other parameters, including NLR, PLR, MLR, SII, and PNI during the observation period (Figure 3B).

### A Nomogram Predicting DFS Based on ALI

Based on the above significant determinants, a predictive nomogram for DFS in patients with CCA was built. As depicted in Figure 3C, a higher total score was associated with a lower DFS rate. The performance of the model was validated internally. The C-index of the nomogram including ALI for predicting DFS was 0.73 (95%CI: 0.67–0.79). The calibration curves showed good agreement between the predicted and actual probability of DFS (Figure 3D). As shown in Figure 3E, the model including ALI provided more predictive power than either the ALI model or the NLR model. The clinical decision curve (Figure 3F) showed the prediction of risk stratification of 1,000 patients using a resampling bootstrap method.

## Discussion

To date, no studies have assessed the clinical implications of the combination of nutrition and immune indicators in patients with CCA. In the present study, ALI, a combined index of immunity and nutrition indices, was identified as an independent risk factor for long-term outcomes in patients with CCA. Additionally, ALI was superior to the previous single index in predicting prognosis in CCA patients. Taken together, these findings suggest that ALI may be an effective prognostic indicator in patients with CCA.

Increasing evidence has demonstrated that nutrition and inflammation are related to tumor development and progression (18, 19). BMI and albumin have been recognized as essential parameters for evaluating the nutritional status of cancer patients (20). Previous studies have shown that preoperative hypoalbuminemia was associated with poor OS in patients with CCA (21, 22). In addition, inflammatory indexes in the peripheral blood, such as NLR (23), MLR (24), PLR (25), SII (26), and PNI (27), have been used as markers of predicting prognosis in CCA patients. Theoretically, the combination of nutrition and inflammatory factors would further increase the prognostication efficacy.

ALI is a novel index calculated from BMI, albumin, and NLR that can conjunctly reflect patients' nutritional and inflammatory status (12). A low ALI score, based on a lower BMI or ALR, or a higher NLR, indicates poor nutritional and high inflammatory status. The clinical utility of ALI as a prognostic indicator has been confirmed in several cancers (13–16). Mandaliya et al. (13) demonstrated that ALI could be used to prognosticate patients with non-small cell lung cancer. The prognostic value of ALI was also verified in patients with colorectal cancer (14). Yin et al. (16) have shown that gastric cancer patients with low ALI showed poorer OS and DFS, and preoperative ALI can be used to predict oncological outcomes in gastric cancer patients. However, few studies focused on the clinical implication of ALI in CCA patients. Thus, the utility of ALI for patients with CCA remains unexplored.

Following these observations, our study demonstrated that CCA patients with low ALI had a significantly worse prognosis than those with high ALI (*P* < 0.001). The 5-year OS and DFS rates were significantly lower in patients with low ALI than patients with high ALI. Using time-dependent ROC analysis, we found that ALI was better at predicting prognosis than other parameters, such as NLR, MLR, PLR, SII, and PNI in terms of OS and DFS. These results indicate that ALI reflects the nutritional and inflammatory status, as well as the potential prognosis of CCA patients.

A valid prognostic model is essential in predicting prognosis and could help facilitate adjuvant therapy and postoperative surveillance in cancer patients (28). Therefore, we constructed a nomogram combined ALI to predict DFS in patients with CCA. Data from the C-index and calibration plots confirmed that the nomogram had a medium prediction accuracy. We also found that nomogram combined ALI was better than the ALI model and the NLR model in predictive accuracy for DFS. Therefore, ALI can supplement the traditional TNM staging system in clinical practice to conduct preoperative risk stratification and prognosis assessment for patients with CCA.

There are several limitations to this study. Firstly, the present study was a retrospective analysis. Second, the sample size was relatively small. Thirdly, ALI was partly based on blood examinations, which could be influenced by many factors, such as the physician's preferences. Finally, the nomogram was validated internally; it should be externally validated in an independent study patient cohort. Therefore, further studies are required to validate the clinical implication of ALI in patients with CCA.

## Conclusion

To our knowledge, this study is the first to assess the clinical utility of ALI in patients with CCA. The results demonstrated that ALI might be an effective indicator for predicting long-term outcomes in patients with CCA. Further studies are warranted to confirm the above findings.

## Data Availability Statement

The original contributions presented in the study are included in the article/Supplementary Material, further inquiries can be directed to the corresponding author/s.

## Ethics Statement

The study protocol was approved by the Institutional Review Board of the First Affiliated Hospital of Fujian Medical University (NO. 2018-053). The patients/participants provided their written informed consent to participate in this study.

## Author Contributions

HW, FD, ML, ZS, ZZ, YC, and PZ designed the study. ZM, SC, CJ, and HQ collected the data and were major contributors in writing the manuscript. All authors read and approved the final manuscript.

## Conflict of Interest

The authors declare that the research was conducted in the absence of any commercial or financial relationships that could be construed as a potential conflict of interest.

## Publisher's Note

All claims expressed in this article are solely those of the authors and do not necessarily represent those of their affiliated organizations, or those of the publisher, the editors and the reviewers. Any product that may be evaluated in this article, or claim that may be made by its manufacturer, is not guaranteed or endorsed by the publisher.
