# Supplementary material for: Use of the Advanced Lung Cancer Inflammation Index as a Prognostic Indicator for Patients With Cholangiocarcinoma
Source: Front Surg. 2022 Jan 27;9:801767. doi: 10.3389/fsurg.2022.801767 (PMC8828638; doi:10.3389/fsurg.2022.801767)
Supplement: Supplementary file 1 [file Data_Sheet_1.PDF]

Supplementary figure 1

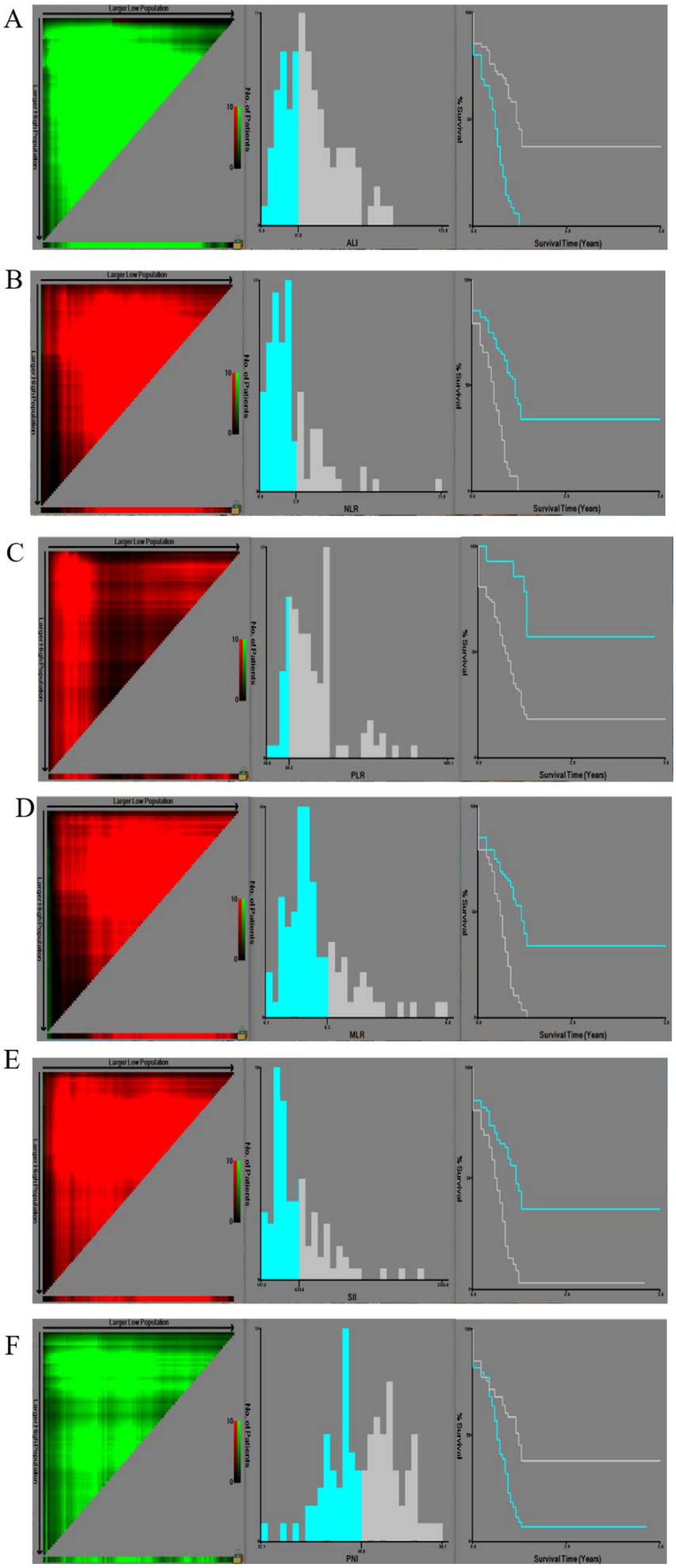

Supplementary Figure 1. Cutoff points for ALI, NLR, PLR, MLR, SII, and PNI counts determined by the X-tile program.

ALI: advanced lung cancer inflammation index, NLR: neutrophil-to-lymphocyte ratio, MLR: monocyte-to-lymphocyte ratio, PLR: platelet-to-lymphocyte ratio, SII: systemic immune-inflammation index; PNI: prognostic nutritional index

Supplementary figure 2

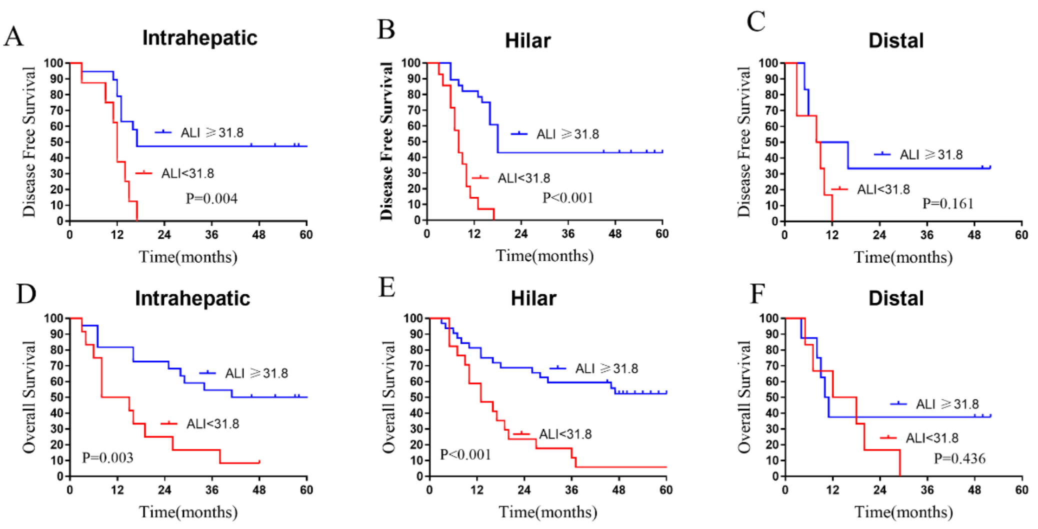

Supplementary Figure 2. Subgroup Kaplan-Meier analysis of ALI according to the location of CCA.

(A) Disease free survival by ALI in intrahepatic CCA patients;

(B) Disease free survival by ALI in hilar CCA patients

(C) Disease free survival by ALI in distal CCA patients

(D) Overall survival by ALI in intrahepatic CCA patients

(E) Overall survival by ALI in hilar CCA patients

(F) Overall survival by ALI in distal CCA patients

CCA: cholangiocarcinomas, ALI: advanced lung cancer inflammation index

Supplementary Table 1. Baseline characteristics in patients with cholangiocarcinoma stratified by tumor location

**Characteristics**	**Intrahepatic (n=34)**	**Hilar (n=49)**	**Distal (n=14)**	***P* value**
Sex (%)				0.635
Male	22 (64.7)	29 (59.2)	7 (50.0)	

Female	12 (35.3)	20 (40.8)	7 (50.0)	
Age (years)	58.9 ± 10.6	61.4 ± 10.1	60.1 ± 11.3	0.557
ASA score (%)				0.852
1	21 (61.8)	33 (67.3)	8 (57.1)	
2	12 (35.3)	13 (26.5)	5 (35.7)	
3	1 (2.9)	3 (6.1)	1 (7.1)	
Preoperative CEA level (%)				0.957
< 5.0 ng/mL	11 (32.4)	16 (32.7)	4 (28.6)	
≥ 5.0 ng/mL	23 (67.6)	33 (67.3)	10 (71.4)	
Preoperative CA19-9 level (%)				0.219
< 37.0 U/mL	16 (47.1)	17 (34.7)	3 (21.4)	
≥ 37.0 U/mL	18 (52.9)	32 (65.3)	11 (78.6)	
Preoperative AFP level (%)				0.376
< 25.0 μg/L	10 (29.4)	16 (32.7)	7 (50.0)	
≥ 25.0 μg/L	24 (70.6)	33 (67.3)	7 (50.0)	
Anemia (%)	11 (32.4)	16 (32.7)	1 (7.1)	0.153
Hypoproteinemia (%)	6 (17.6)	11 (22.4)	1 (7.1)	0.424
CRP (%)				0.425
< 10.0 mg/L	32 (94.1)	42 (85.7)	13 (92.9)	
≥ 10.0 mg/L	2 (5.9)	7 (14.3)	1 (7.1)	
PCT (%)				0.688
< 0.06 ng/mL	29 (85.3)	41(83.7)	13 (92.9)	
≥ 0.06 ng/mL	5 (14.7)	8 (16.3)	1 (7.1)	
BMI (%)				0.549
<18 kg/m^2	1 (2.9)	0	1 (7.1)	
18-24 kg/m^2	22 (64.7)	32 (65.3)	8 (57.1)	
≥ 24 kg/m^2	11 (32.7)	17 (34.7)	5 (35.7)	
Tumor differentiation (%)				0.074
Well to moderately differentiated	21 (61.8)	40 (81.6)	12 (85.7)	
Poorly differentiated and others	13 (38.2)	9 (18.4)	2 (14.3)	
Postoperative complications (%)	38 (39.2)	12 (34.3)	26 (42.6)	0.517
Pathological T stage (%)				0.327
1	1 (2.9)	2 (4.1)	0	
2	10 (29.4)	9 (18.4)	7 (50.0)	
3	20 (58.6)	30 (61.2)	5 (35.7)	

4	3 (8.8)	8 (16.3)	2 (14.3)	
Pathological N stage (%)				0.832
Negative	21 (61.8)	27 (55.1)	8(57.1)	
Positive	13 (38.2)	22 (44.9)	6 (42.9)	
Pathological M stage (%)				0.727
0	27 (79.4)	42 (85.7)	12 (85.7)	
1	7 (20.6)	7 (14.3)	2 (14.3)	
Pathological T stage (%)				0.148
1	1 (2.9)	2 (4.1)	0	
2	7 (20.6)	7 (14.3)	7 (50.0)	
3	19 (55.9)	33 (67.3)	5 (35.7)	
4	7 (20.6)	7 (14.3)	2 (14.3)	
Nerval invasion (%)	19 (55.9)	23 (46.9)	8 (57.1)	0.654

ALI: advanced lung cancer inflammation index; ASA: American Society of Anesthesiologists; CEA: carcinoembryonic antigen; CA19-9: carbohydrate antigen 19-9; AFP: α-fetoprotein; CRP: C-reactive protein; PCT: procalcitonin; BMI: body mass index
